# Dilution of whisky – the molecular perspective

**DOI:** 10.1038/s41598-017-06423-5

**Published:** 2017-08-17

**Authors:** Björn C. G. Karlsson, Ran Friedman

**Affiliations:** 1 Physical Pharmacy Laboratory, Kalmar, SE 391-82 Sweden; 2 Computational Chemistry & Biochemistry Group, Kalmar, SE 391-82 Sweden; 3 Linnæus University Centre for Biomaterials Chemistry, Kalmar, SE 391-82 Sweden

## Abstract

Whisky is distilled to around 70% alcohol by volume (vol-%) then diluted to about 40 vol-%, and often drunk after further slight dilution to enhance its taste. The taste of whisky is primarily associated with amphipathic molecules, such as guaiacol, but why and how dilution enhances the taste is not well understood. We carried out computer simulations of water-ethanol mixtures in the presence of guaiacol, providing atomistic details on the structure of the liquid mixture. We found that guaiacol is preferentially associated with ethanol, and, therefore, primarily found at the liquid-air interface in mixtures that contain up to 45 vol-% of ethanol. At ethanol concentrations of 59 vol-% or higher, guaiacol is increasingly surrounded by ethanol molecules and is driven to the bulk. This indicates that the taste of guaiacol in the whisky would be enhanced upon dilution prior to bottling. Our findings may apply to other flavour-giving amphipathic molecules and could contribute to optimising the production of spirits for desired tastes. Furthermore, it sheds light on the molecular structure of water-alcohol mixtures that contain small solutes, and reveals that interactions with the water may be negligible already at 89 vol-% of ethanol.

## Introduction

Whisky is a spirit that is produced in an extended process consisting of distillation of fermented grains, ageing and dilution. It is through this process that the distinctive taste of whisky develops. Distilled malt whiskies typically contain around 70% alcohol by volume (vol-%) before it is aged in barrels for at least three years. Some alcohol evaporates during the maturation resulting in an alcohol content of 55–65 vol-% of cask-strength whisky. Before bottling, the whisky is diluted to around 40 vol-% by the addition of water^1^, which changes the taste significantly. Whisky enthusiasts often also add a few drops of water to the spirit before drinking in order to further enhance the taste. Apart from water and alcohols, whiskies contain different organic compounds that contribute to their taste^2^. Many whiskies, especially those that are made on the Scottish island of Isley, have a typical smoky taste that develops when malted barley is smoked on peat fire. Chemically, the smoky flavour is attributed to phenols, and in particular guaiacol, which is much more common in Scottish whiskies than in American or Irish ones^3, 4^. Guaiacol is a small and mostly hydrophobic molecule that is able to interact with polar solvents via hydrogen-bonding and polar-aromatic interactions. Higher concentrations of guaiacol have been found in Scottish whiskies than in American and Irish ones. The concentration of guaiacol was found by GC/MS to be 3.7–4.1 mg L^−1^, or about 3.2·10^−5^ M in two undisclosed Scottish whiskies^5^. It is likely that the concentration of guaiacol in Isley whiskies is even higher. Yet, how diluting whisky with water affects its taste is not clear.

Spectroscopic studies have previously revealed that alcohol and water undergo incomplete mixing^6–9^. Concentration-dependent variations in the liquid structure were evident from ultrasonic measurements^10^ and X-ray absorption spectroscopic studies^11^ of water-alcohol mixtures. Further investigations suggested that the behaviour of such mixtures could be divided into three regions of low, middle and high molar fraction of alcohol^12^. Molecular dynamics (MD) simulations showed that water and short-chain alcohols in liquid mixtures separate into clusters^13^. The dynamics of these clusters was first studied by MD and non-equilibrium simulations, revealing that such clusters do not move by collective diffusion^14^. Recently, vibrational spectroscopy and theoretical analysis showed that aggregation of short chain alcohols in mixtures that contain up to 4 M alcohol in water are, by and large, a random process^15^. Simulations of surface interfaces of water-ethanol mixtures have also been performed in the past^16–18^ and shed light on the structure and dynamics of the alcohol-air interface and on the mixing of alcohol and water in the presence of an additional surface. Simulations and potential of mean force calculations of droplets of water-alcohol mixtures showed a clear preference of the alcohols to the air-water interface, which was shown to be primarily an enthalpic effect^19^. Furthermore, decomposition of the solvation energy of ions and non-polar solutes at the solvent-air interface was performed by applying linear response theory^20^. Interestingly, alkyl-halides were also shown to be adsorbed at the air-water interface, where they were partially solvated by water^21^.

Despite the growing knowledge of the nature of water-alcohol mixtures on a molecular level, much less is known on the interaction of water, alcohol and small solutes. In particular, the nature of the interaction between the solvent and taste-carrying molecules, such as guaiacol, is not known. To address this gap, we used MD simulations to study the distribution of guaiacol in water-alcohol mixtures of different concentrations. Our simulations revealed that guaiacol is present at the air-liquid interface at ethanol concentrations that correspond to the alcohol content of bottled or diluted whiskies. Because the drink is consumed at the interface first, our findings help to understand why adding water to whisky helps to enhance its taste. A molecular understanding of the nature of taste compounds in water-alcohol mixtures allows for optimizing the taste of alcoholic spirits. Moreover, such an understanding can contribute to the development of stock solution drug formulations. Indeed, guaiacol is one of the active compounds in a pharmaceutically active anti-cough oral solution, Pulmo Bailley®, in a mixture with water and a short polyol alcohol (glycerol).

## Computational Methods

**Figure 1:**
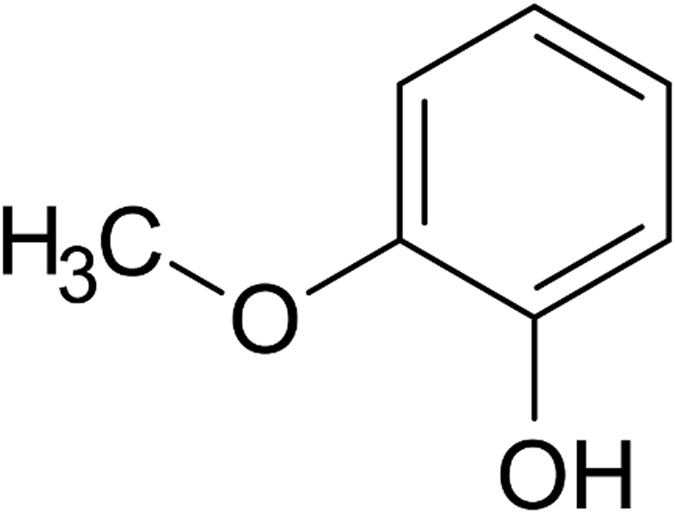
2-methoxy phenol, guaiacol.

Single molecules of the solute guaiacol (2-methoxy phenol), Fig. 1, and ethanol (EtOH), were initially built in Avogadro^22^, pre-minimised using MMFF94^23^ and further optimised in Gaussian09 (v. 09, revision A.02, Gaussian, Inc., Wallingford, USA)^24^. The opt = tight convergence criteria was used as implemented in the software at the HF/6–31G* level of theory as recommended for use with the Amber family of force fields^25^. Atomic partial charges were derived through the restrained electrostatic potential (RESP) procedure^26^. Molecular coordinates representing a series of EtOH-water mixtures were built in PACKMOL^27^ with molar ratios of EtOH (*X*_EtOH_) ranging from 0 to 1.0, using 0.1 increments, both in the absence and presence of the solute guaiacol. In the Supplementary Tables S1 and S2 more details are presented with regards to the mixture design, the starting box coordinates and the coordinates obtained after 10 ns of simulation.

All-atom MD simulations were performed using the Amber software (v. 10, USCF, San Francisco, USA)^28, 29^ together with the Amber12SB and the compatible GAFF^30^ force fields, which were developed for organic molecules. The SPC/E^31^ water model, which includes the average effect of polarisation in polar liquids, was used because the model is known to correctly reproduce the density, radial distribution function and self-diffusion constant of water^32^. Moreover, the model has been shown to be adequate for simulations at the air-water interface^33, 34^. In order to test the validity of the adapted parameters, a series of reference simulations of guaiacol, the pure solvents, and of the water-EtOH mixtures in the absence of guaiacol were performed. Simulations at the melting temperature of guaiacol (T = 301.15 K) resulted in enthalpy of vaporisation of 67.6 kJ mol^−1^, which is in good agreement with the experimental value of 60.4 kJ mol^−1^ ^35^. The corresponding value for EtOH at 298.15 K was calculated to 46.1 kJ mol^−1^, which is also in agreement with the experimental value of 42.3 kJ mol^−1^ ^35^. The densities of the liquid-phases of guaiacol and ethanol were calculated to 1.15 g cm^−3^ (301.15 K) and 0.80 g cm^−3^ (298.15 K), which is in general agreement with the experimental data^36^ of 1.11 and 0.785 g cm^−3^, respectively.

Calculations of water-EtOH excess volumes for mixtures in the absence of guaiacol reproduced the experimental finding that solutions with a molar fraction of *X*_*EtOH*_ = 0.3–04 (59–69 vol% of EtOH) give rise to the greatest deviation from ideal behaviour, see Supplementary Table S3 and Figure S1. As in earlier simulations reported by others^37^, however, the differences in excess volume were higher in the experiment than in the simulations. The concentration of EtOH in each mixture is also presented as volume-% (vol-%). The approximate value of vol-% of ethanol in each mixture was obtained from a calculation of the molecular volume of ethanol, as explained in Table S3 and added to the text to aid in the interpretation of data.

Likewise, estimations of the static dielectric constant for each water-EtOH mixture through calculations of the fluctuation of the total dipole moment (<M^2^>-<M>^2^)^38, 39^ reproduced the decrease of the dielectric constant as observed in dielectric relaxation experiments^40^. The absolute value of the obtained dielectric constant, however, was lower than in the experiments, see Supplementary Figure S2. Calculations of the Stokes-Einstein self-diffusion rates of water and ethanol in each mixture, both in absence and presence of guaiacol, revealed no influence of the presence of the solute in the mixtures, see Supplementary Table S4.

### Bulk-phase simulations

Each system was initially energy minimised to remove unfavourable van der Waals contacts using a total of 10000 steps. These were divided into 5000 steepest descent steps followed by 5000 steps of conjugate gradient minimisation. After the energy minimisation, velocities for all atoms were randomly assigned from a Maxwell-Boltzmann distribution of velocities at T = 298.15 K, and the systems were simulated for 100 ps under NVT conditions (*i.e*. constant number of particles, volume and temperature). An additional simulation of 500 ps was then conducted at a condition of constant number of particles, a pressure of 1 bar and a temperature of T = 298.15 K. The production phase data were collected for each system from 10 ns of simulation at NPT conditions.

During all simulations, the temperature was held constant using Langevin dynamics with a collision frequency set to 1.0 ps^−1^. The NPT simulation steps were conducted using an isotropic pressure scaling with the Berendsen barostat and a pressure relaxation constant τ_p_ of 2.0 ps. All bonds to hydrogen were constrained using the SHAKE algorithm^41^ that allowed a time step set to 0.002 ps. Periodic boundary conditions were applied in all directions using a 12-Å non-bonded interaction cut-off. Long-range electrostatics were treated using the particle mesh Ewald (PME) summation method, and long-range van der Waals interactions were corrected using a continuum model correction of both energy and pressure. Data was collected every 2 ps.

### Liquid-air interface simulations

Boxes representing molecular coordinates of the series of water-EtOH-guaiacol mixtures that were obtained after 10 ns of simulations were inserted and centred as a liquid-slab in new boxes. Each of these boxes had the same *x* and *y* dimensions as before, whereas the *z*-axis was elongated by half its dimension at its positive and negative sides. The mixtures were then subsequently simulated at conditions of constant volume for further 50 ns typically collecting data every 10 ps.

### Analysis

Spatial distribution functions (SDFs) were calculated using the Grid command implemented in the AmberTools suite of programs (v. 15, USCF, San Francisco, USA). The positions of selected atoms of water and EtOH around guaiacol were binned from root-mean-square coordinate fit frames over all guaiacol atoms at intervals of 10 ps intervals for 50 ns liquid-air interface simulations, and at intervals of 2 ps for 10 ns bulk-phase simulations. A grid spacing of (0.5)^3^ Å^3^ and a box size of 100^3^ Å^3^ were used in all calculations.

Graphical representations of the occupancy of selected solvent atoms around the solute guaiacol were shown using either 3 or 8 times the contour value expected for the bulk density of the pure solvents. The values used as reference bulk densities (Density_Bulk_) of water and ethanol in all mixtures were calculated to 20.9 (0.03346 atoms/Å^3^ × 0.125 Å^3^ × 5000 frames) and 6.5 (0.01046 × 0.125 Å^3^ × 5000 frames), respectively.

One-dimensional densities were calculated with the Density Profile Tool^42^ as implemented in VMD (version 1.9.1, University of Illinois at Urbana-Champaign, USA)^43^.

All z-axis atomic densities were calculated as the mean ± the standard deviation over the total simulation time of 50 ns with the centre of the box along the *z*-axis set to *z* = 0, taking into account each density value along the *z-*axis by using absolute values of the *z*-axis coordinates. For reasons of clarity, the final density profile has been symmetrized and shows both negative and positive values of *z*.

When error estimates are reported, values are presented as the mean ± standard deviation from five separate 30 ns blocks of data covering the total simulation time of 50 ns (0–30 ns, 5–35 ns, 10–40 ns, 15–45 ns, and 20–50 ns).

## Results

### EtOH molecules demonstrate surfactant-like behaviour

**Figure 2:**
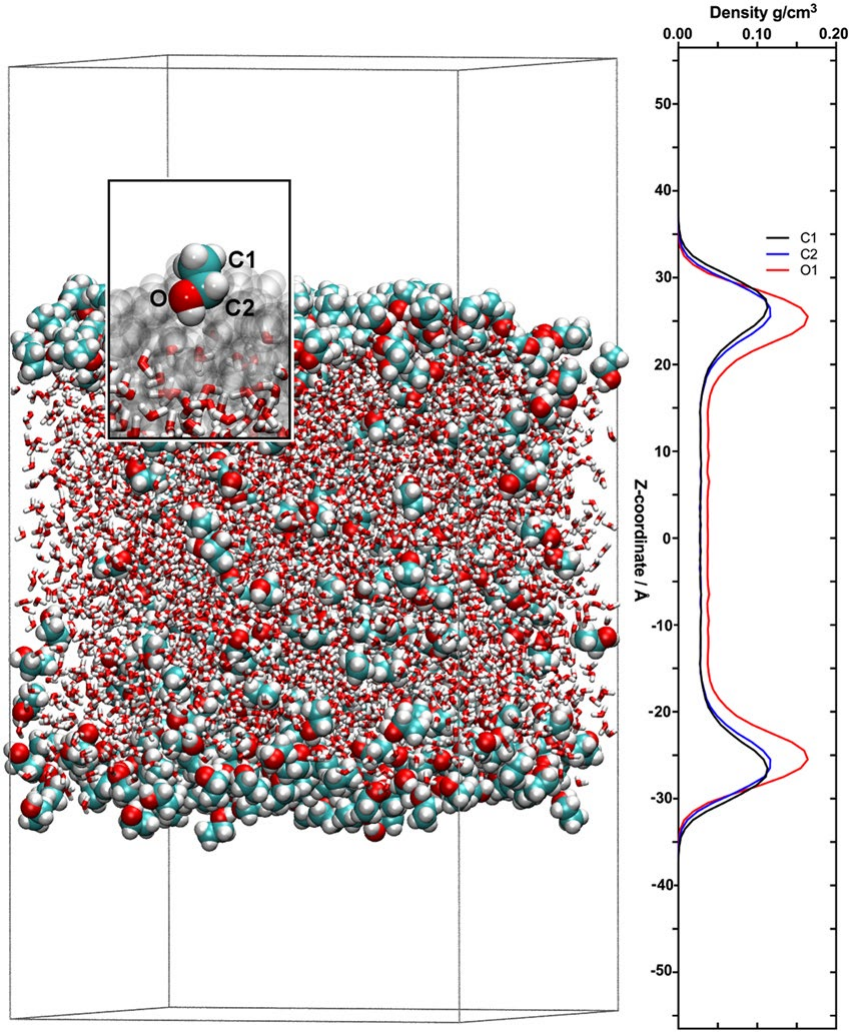
Preferred distribution of EtOH molecules. Snapshot of an all-atom MD simulation of a 27 vol-% EtOH mixture with a single guaiacol molecule. EtOH molecules are shown in van der Waals representations, and the water molecules are shown in licorice representations. The carbon atoms are displayed in turquoise, hydrogen atoms in white and oxygen atoms in red. The *z*-axis atomic mass density distribution of the carbon (C1 and C2) and oxygen (O) atoms of EtOH is also shown. The outwards-orientation of the ethyl groups suggests that EtOH molecules localised at the liquid-air interface behave like a surfactant. In the insert, a single molecule of EtOH is shown in van der Waals representation, the remaining EtOH molecules are displayed in grey transparent surface representations and water molecules in licorice representations.

In order to obtain a molecular understanding of water-EtOH mixtures, all-atom MD simulations of a series of water-EtOH-guaiacol liquid-air interface mixtures were performed. We found that water and EtOH demonstrate non-ideal mixing behaviour. In particular, we observed a microscopic phase separation occurred in such mixtures: At lower concentrations, EtOH was found to be preferentially located and packed at the liquid-air interface, with the ethyl groups oriented towards the gas-phase, as shown in Fig. 2 for a 27 vol-% EtOH mixture.

### Guaiacol co-localises preferentially at the liquid-air interface in EtOH concentrations of 45 vol-% or lower

**Figure 3:**
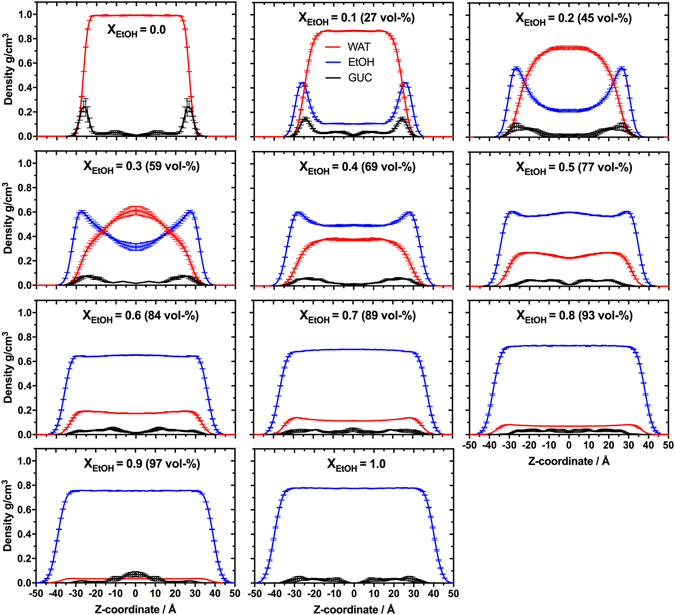
Molecular distributions of guaiacol, water and EtOH along the z-axis for different EtOH concentrations. The figure shows the results of liquid-air interface simulations over 50 ns of the mass density distributions of water (WAT, red), guaiacol (GUC, black) and EtOH (blue) in a mixture. For clarity, calculated mass densities were averaged along the z-axis and the mass density of guaiacol was multiplied by a factor of 50. Values are here presented as the mean ± standard deviation from five separate 30 ns blocks of data covering the total simulation time of 50 ns (0–30 ns, 5−35 ns, 10–40 ns, 15–45 ns, and 20–50 ns).

In the next step, the preferred location of guaiacol in the EtOH-water mixtures was investigated. Therefore, the molecular distribution of guaiacol and the solvent molecules along the surface normal (*z*-axis) was studied. Our results demonstrate non-ideal behaviour of the mixtures with different EtOH contents, Fig. 3. Guaiacol prefers to be co-localised at the interface in pure water and in mixtures with EtOH concentrations of up to ca. 45 vol-%. Increasing the EtOH content of the mixture to 59 vol-% resulted in a translocation of guaiacol towards the bulk-phase of the mixture. This shift in guaiacol distribution indicates that EtOH provides a molecular environment that is preferred by guaiacol. Based on this observation, we suggest that the dilution of a water-EtOH mixture initially containing ca. 59 vol-% EtOH (*e.g*., cask strength whisky)^1^ would lead to an increasing probability of finding guaiacol at the liquid-air interface with decreasing EtOH content. Although no evaporation of guaiacol from the interface into the gas-phase could be observed during the time-frame of simulation (50 ns) it is reasonable to assume that the increase in the probability of finding guaiacol at the interface leads to a higher probability of its evaporation. Hence, the addition of water at this breaking point will result in an increased probability of finding guaiacol at the liquid-air interface, which likely influences both taste and smell of the whisky. Of note, it has recently been shown that surface-active molecules are depleted from the liquid-air interface when the concentration of ethanol is increased in ethanol-water mixtures, which corroborates our findings^44^.

### Water-ethanol mixtures are heterogeneous

**Figure 4:**
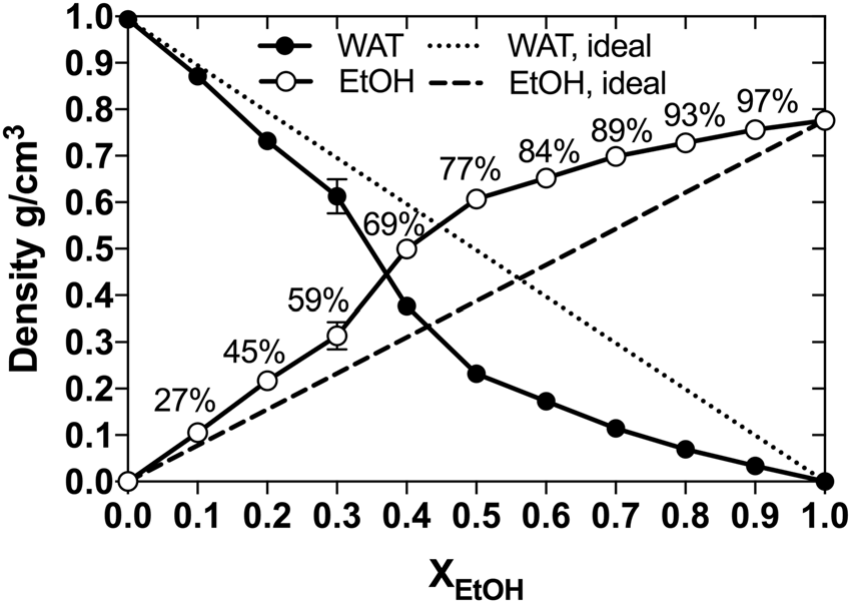
Bulk density of solvents. The calculated (circles) bulk densities (at z = 0) of water (WAT, filled circles) and EtOH (empty circles) are shown as functions of EtOH concentration. The dots were connected with lines as guides for the eye. The non-ideal mixing behaviour is evident by comparison with the theoretical densities (dashed lines) of water and EtOH in an ideal mixture. Values are here presented as the mean ± standard deviation from five separate 30 ns blocks of data covering the total simulation time of 50 ns (0–30 ns, 5–35 ns, 10–40 ns, 15–45 ns, and 20–50 ns).

To shed further light on the influence of the heterogeneity of water-EtOH mixtures on the liquid-phase distribution of guaiacol, calculations of the density of each solvent at the centre of the liquid mixture (*z* = 0), here defined as the liquid bulk region, were performed, Fig. 4. In line with our previous observations, EtOH was found to prefer the liquid-air interface and, therefore, an increase in the concentration of EtOH in the mixtures of EtOH resulted in an increase in the bulk concentration of EtOH compared to the expected concentration in an ideal mixture. At low EtOH contents, the alcohol was found at the interface. With increasing alcohol content, the interface becomes increasingly packed, and the EtOH molecules were observed to enter the bulk phase and form clusters to minimise their contacts to water. The largest deviation from complete mixing was observed at 59–77 vol-%, Fig. 4, which coincides with the transition of guaiacol from the interface to bulk-phase (as evident from Fig. 3).

**Figure 5:**
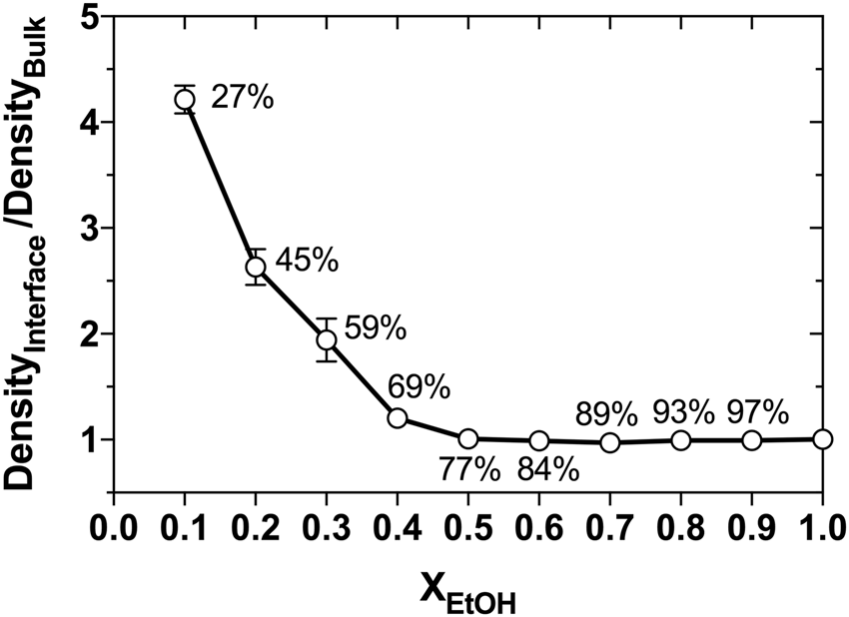
Interface accumulation of EtOH. The accumulation of EtOH (open circles) at the liquid-air interface (here described as the ratio of the mass densities for EtOH at the interface (z-axis maximum density) and the mass bulk density at z = 0 (Density_Interface_/Density_Bulk_), as a function of the EtOH concentration. Values are here presented as the mean ± standard deviation from five separate 30 ns blocks of data covering the total simulation time of 50 ns (0–30 ns, 5–35 ns, 10–40 ns, 15–45 ns, and 20–50 ns).

An analysis of the relative density of EtOH located at the interface compared to the density in the bulk, shown in Fig. 5, revealed that an increase in the EtOH content from 27–77 vol-% resulted in an almost linear decrease in the relative amount of EtOH molecules found at the interface, whereas the relative amount of EtOH at the interface was observed to be constant and low at concentrations above 77 vol-%.

### The distribution of the solvents around guaiacol

**Figure 6:**
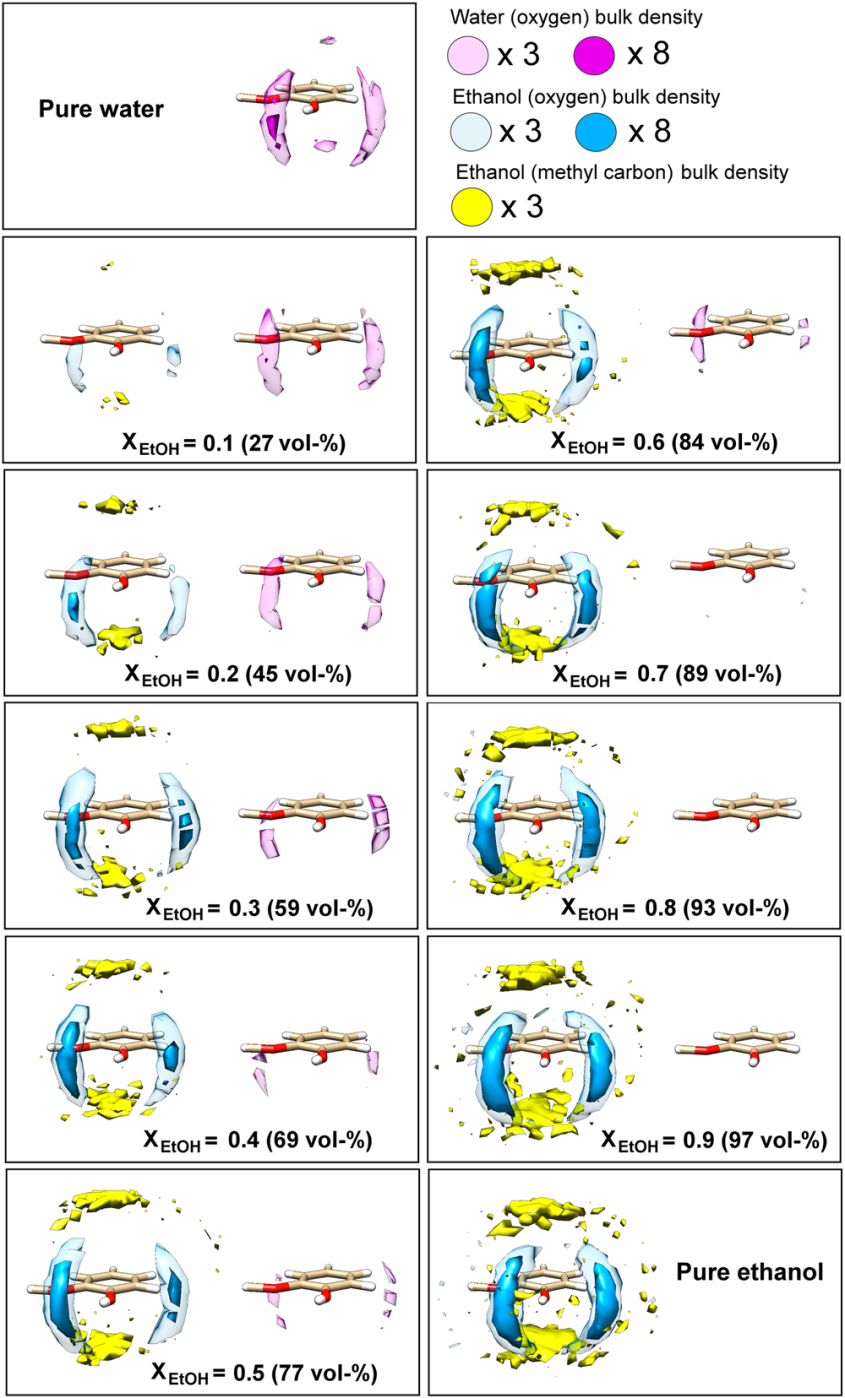
Solvent affinity of water and EtOH for guaiacol. Atomic spatial distribution functions (SDFs) of water oxygen and EtOH oxygen and methyl carbon components around a single guaiacol molecule in liquid-air interface mixtures of varying EtOH content. In each panel, the water and EtOH guaiacol solvation distributions are shown on the right- and left-hand side, respectively. For better visibility, the atomic densities of selected atoms of the liquids, *i.e*. water and ethanol oxygen, and the methyl carbon of ethanol, are presented using either three or eight times the water or ethanol bulk densities.

In order to estimate the relative affinity of the solvent species for guaiacol in the mixtures, we performed a series of spatial distribution functions (SDFs) representing probabilities of finding water and EtOH molecules around guaiacol throughout 50 ns of liquid-air interface simulations, Fig. 6. Both EtOH and water oxygen atoms were found to prefer the phenol ring of guaiacol. Notably, increasing the concentration of EtOH in the mixtures resulted in a strong increase in the occupancy of the oxygen atoms of EtOH at a position between the phenolic and methoxy groups of guaiacol. At this location, the hydroxyl groups of water and EtOH could interact through hydrogen bonding with either moiety. This hydrogen-bonding pattern appears to have a big influence on the structure of the first-solvation shell, as evident from Fig. 6. The methyl group of EtOH was preferentially found above and below the phenyl ring of guaiacol. This observation indicates that a stacking interaction between the ethyl group of EtOH and the phenyl ring of guaiacol occurred for all water-EtOH mixtures studied.

**Figure 7:**
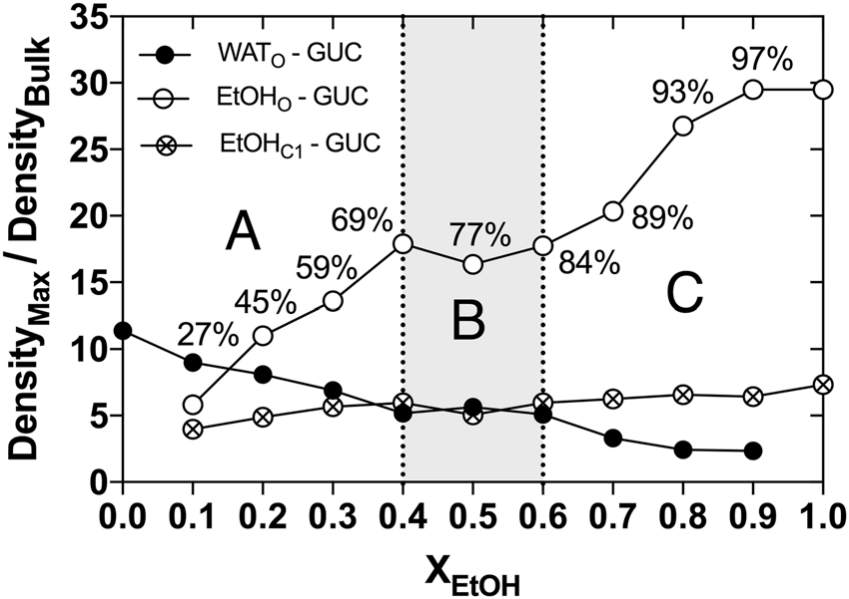
Maximum occupancy (Density_Max_/Density_Bulk_) for the components of the solvents around guaiacol (GUC). The maximum occupancy here shown for water (filled circles) and EtOH (open circles) oxygen atoms, WAT_O_ and EtOH_O_, respectively, and the EtOH methyl carbon, EtOH_C1_, (crossed circles) was calculated by dividing the observed maximum atomic density, Density_Max_ with the expected bulk atomic density value, Density_Bulk_, on the basis of the spatial distribution functions presented in Fig. 6. As a guide for the eye, the dots are connected with lines. EtOH concentration ranges that are discussed in the paper have been marked out with the letters (**A**–**C**).

A quantitative analysis of the maximum atomic densities of each of the solvent-guaiacol contacts revealed differences with respect to the EtOH content, as presented in Fig. 7. The observed effects can be divided into three groups: Between 0–69 vol-% (region A) of EtOH, increasing EtOH concentrations resulted in a competition between EtOH and water for hydrogen bonding at the phenolic and methoxy functionalities of guaiacol. Between 69–84 vol-% of EtOH (region B), the affinity of both EtOH and water to guaiacol was observed to be largely unchanged. This suggests that in this range of EtOH content, the solvent shell of guaiacol contained both water and EtOH. Between 84–100 vol-% of EtOH (region C), small changes in the EtOH concentration resulted in noticeable shifts in the affinity of the solvents to guaiacol, *i.e*. a decrease of water affinity and an increase of EtOH affinity to guaiacol. This implies that in this range of high EtOH concentrations, the first solvation shell is comprised only of EtOH. The corresponding atomic SDFs analysis of bulk-phase simulations of the water-EtOH solvation of guaiacol resulted in similar profiles, as shown in Supplementary Figures S3 and S4. These results suggest that the introduction of a liquid-air interface does not influence the extent of contacts between guaiacol and the solvent molecules.

## Discussion

### Dilution of cask-strength whisky improves its taste by increasing the propensity of taste compounds at the liquid-air interface

We carried out MD simulations of guaiacol-water-alcohol solutions of various EtOH concentrations to understand why the taste of whisky changes upon the addition of water. The simulations revealed that EtOH and water mix non-ideally generating clusters of EtOH molecules. The solvation of guaiacol in water or EtOH was assisted by the formation of hydrogen bonds to its two oxygen atoms. For EtOH, an additional interaction with guaiacol was found between the short alkyl chain of EtOH and the aromatic ring of guaiacol. Therefore, guaiacol was preferentially associated with EtOH and thus, at low EtOH concentrations, more likely to be present at the liquid-air interface than in the bulk. In a glass of whisky, which typically exhibits alcohol concentrations of ca. 45 or 27 vol-% if diluted, guaiacol will thus be found near the liquid surface, where it greatly contributes to both smell and taste of the spirit. At cask-strength concentrations of 59 vol-% or higher, EtOH interacts more strongly with the guaiacol above and below the ring-plane, and guaiacol is therefore driven into the solution. It is therefore reasonable to assume that the taste of guaiacol (and other amphipathic, semi-volatile compounds) is less pronounced at high alcohol concentrations, which explains why dilution of cask-strength whiskies results in a change in the sensory effects of the whisky. Although the target of our studies is the solution behaviour of guaiacol in various water-ethanol mixtures it cannot be ruled out that a change in the concentration of ethanol in the mixtures (either by a dilution or evaporation) can also change the taste and smell of a whisky.

Like guaiacol, many flavour compounds that are found in alcoholic liquors are small organic molecules that are slightly volatile and amphipathic. Examples include vanillin, ethyl acetate and limonene. This may explain why spirits containing amphipathic molecules such as guaiacol are most often sold at concentrations of 30–50 vol-%. More hydrophobic compounds such as anethole, which contributes to the characteristic flavour of aniseed drinks, are soluble almost only in EtOH^45^. Indeed, unlike whiskies, aniseed-flavoured spirits are usually diluted heavily prior to drinking, *e.g*. two parts of water per one part of spirit, which creates an emulsion and increases the perception of anethole.

### Why whisky may taste differently when diluted in the glass

When whisky is diluted from 45 to 27 vol-% of EtOH, guaiacol loses approximately 53% of its contacts with EtOH in the first solvation shell, Figs 6 and 7. This decrease in the number of contacts with EtOH is not matched with a similar increase in the number of contacts to water as the density of water in guaiacol’s solvation shell increases only by 11%. At the same time the interface density of EtOH increase by ~66%, Fig. 5. Since the probability of finding guaiacol at the liquid-air interface increases together with that of EtOH while at the same time guaiacol loses contacts to EtOH, it is now more probable for guaiacol to evaporate and contribute to the aroma of whisky. Thus, the taste of guaiacol and similar compounds will be more pronounced when whisky is further diluted in the glass. This taste-enhancement is counteracted by the dilution of guaiacol’s concentration. Overall, there is a fine balance between diluting the whisky to taste and diluting the whisky to waste. This balance will depend on the concentration and types of taste compounds that are characteristic for each whisky. Similar considerations can be used to optimise the alcohol concentration of other spirits including gin, rum and brandy.

### Solute behaviour in mixtures of water and simple alcohols

This study provided important details on the structure of water-EtOH mixtures and their interactions with a small amphipathic solute. Although the solute was chosen to represent a typical taste compound, the molecular understanding of its distribution at the air-water interface as a function of EtOH concentration is not limited to beverages. Some pharmaceutical formulations, such as cabazitaxel, are stored in solutions containing EtOH prior to the addition of water. It may be desired to estimate if such compounds are more prevalent at the air-water interface prior to injecting them into human or animal subjects.

Furthermore, one of the findings of this study was that guaiacol (at the concentration studied, about 5 mM) is almost completely solvated by EtOH in solutions of 89 vol-% or higher. This consideration may be relevant for storage of compounds that should be shielded from water, *e.g*. because they may be oxidized.

## Electronic supplementary material

Supporting information
