# Author Correction: Dilution of whisky – the molecular perspective

**DOI:** 10.1038/s41598-018-34169-1

**Published:** 2018-11-01

**Authors:** Björn C. G. Karlsson, Ran Friedman

**Affiliations:** 1 Physical Pharmacy Laboratory, Kalmar, SE 391-82 Sweden; 2 Computational Chemistry & Biochemistry Group, Kalmar, SE 391-82 Sweden; 3 Linnæus University Centre for Biomaterials Chemistry, Kalmar, SE 391-82 Sweden

Correction to: *Scientific Reports* 10.1038/s41598-017-06423-5, published online 17 August 2017

This Article contains errors in Figure 4, where the ideal densities of Ethanol and Water in the mixtures are incorrectly represented. The correct Figure 4 appears below.

**Figure 4:**
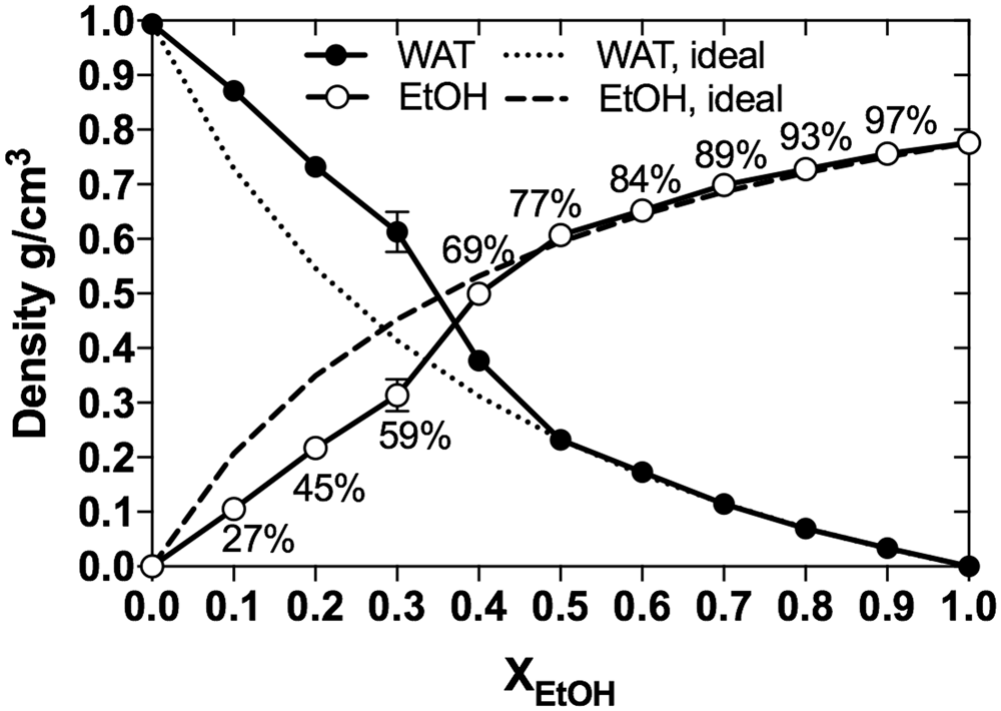
Bulk density of solvents. The calculated (circles) bulk densities (at z = 0) of water (WAT, filled circles) and EtOH (empty circles) are shown as functions of EtOH concentration. The dots were connected with lines as guides for the eye. The non-ideal mixing behaviour is evident by comparison with the theoretical densities (dashed lines) of water and EtOH in an ideal mixture. Values are here presented as the mean ± standard deviation from five separate 30 ns blocks of data covering the total simulation time of 50 ns (0–30 ns, 5–35 ns, 10–40 ns, 15–45 ns, and 20–50 ns).

Additionally, in the Results section under the subheading ‘Water-ethanol mixtures are heterogeneous’,

“The largest deviation from complete mixing was observed at 59-77 vol-%, Fig. 4, which coincides with the transition of guaiacol from the interface to bulk-phase (as evident from Fig. 3).”

should read:

“The largest deviation from complete mixing was observed at approximately 59 vol-%, Fig. 4, which coincides with the transition of guaiacol from the interface to bulk-phase (as evident from Fig. 3).”

