# Supplementary material for: Dilution of whisky – the molecular perspective
Source: Sci Rep. 2017 Aug 17;7:6489. doi: 10.1038/s41598-017-06423-5 (PMC5561096; doi:10.1038/s41598-017-06423-5)
Supplement: Supplementary file 1 — Supporting information [file 41598_2017_6423_MOESM1_ESM.doc]

***Supplementary data for:***

**Dilution of whisky – the molecular perspective**

**Björn CG Karlsson,^1,3^* and Ran Friedman^2,3^**

*^1^Physical Pharmacy Laboratory, ^2^Computational Chemistry & Biochemistry Group, ^3^Linnæus University Centre for Biomaterials Chemistry, SE 391-82 Kalmar, Sweden.*

* Correspondence to:

Björn C.G. Karlsson

Tel: +46 - 480 446740

Fax: +46 - 480 446262

E-mail: [bjorn.karlsson@lnu.se](mailto:bjorn.karlsson@lnu.se)

**Table of Contents Name Page**

Mixture design – in the absence of guaiacol Table S1 S2

Mixture design – in the presence of guaiacol Table S2 S2

Bulk-phase simulation data Table S3 S3

Excess volume Figure S1 S3

Static dielectric constant Figure S2 S4

Solvent self-diffusion rates Table S4 S4

Bulk-phase spatial distribution functions Figure S3 S5

Maximum bulk-phase solvent atomic density Figure S4 S6

**Mixture design – in the absence of guaiacol**

| **Table S1.** Number of atoms and molecules and box dimensions for systems prepared in the absence of the solute guaiacol (GUC). | | | | | | | | |
| --- | --- | --- | --- | --- | --- | --- | --- | --- |
|
|  | Number of Molecules | | |  |  |  | x,y,z (Å) | |
| X_EtOH_ | Water | Ethanol | GUC |  | Atoms |  | t=0 | t=10 ns |
| 0 | 5000 | 0 | - |  | 15000 |  | 58.6×58.6×58.6 | 53.1×53.1×53.1 |
| 0.1 | 4500 | 500 | - |  | 18000 |  | 63.6×63.6×63.6 | 56.4×56.4×56.4 |
| 0.2 | 4000 | 1000 | - |  | 21000 |  | 65.6×65.6×65.6 | 59.6×59.6×59.6 |
| 0.3 | 3500 | 1500 | - |  | 24000 |  | 68.6×68.6 ×68.6 | 62.6×62.6×62.6 |
| 0.4 | 3000 | 2000 | - |  | 27000 |  | 70.6 ×70.6×70.6 | 65.2×65.2×65.2 |
| 0.5 | 2500 | 2500 | - |  | 30000 |  | 73.6 ×73.6×73.6 | 67.7×67.7×67.7 |
| 0.6 | 2000 | 3000 | - |  | 33000 |  | 75.6×75.6×75.6 | 70.0×70.0×70.0 |
| 0.7 | 1500 | 3500 | - |  | 36000 |  | 78.5×78.6×78.6 | 72.2×72.3×72.3 |
| 0.8 | 1000 | 4000 | - |  | 39000 |  | 80.5×80.5×80.5 | 74.3×74.3×74.3 |
| 0.9 | 500 | 4500 | - |  | 42000 |  | 82.5×82.5×82.5 | 76.3×76.3×76.3 |
| 1.0 | 0 | 5000 | - |  | 45000 |  | 84.5×84.5×84.5 | 78.2×78.2×78.2 |
|  | | | | | | | | |

**Mixture design – in the presence of guaiacol**

| **Table S2**. Number of atoms and molecules and box dimensions for systems prepared in the presence of the solute guaiacol (GUC). | | | | | | | | |
| --- | --- | --- | --- | --- | --- | --- | --- | --- |
|
|  | Number of Molecules | | |  |  |  | x,y,z (Å) | |
| X_EtOH_ | Water | Ethanol | GUC |  | Atoms |  | t=0 | t=10 ns |
| 0 | 5000 | 0 | 1 |  | 15017 |  | 58.6×58.6×58.6 | 53.2×53.2×53.2 |
| 0.1 | 4500 | 500 | 1 |  | 18017 |  | 63.6×63.6×63.6 | 56.5×56.5×56.5 |
| 0.2 | 4000 | 1000 | 1 |  | 21017 |  | 65.6×65.6×65.6 | 59.7×59.6×59.7 |
| 0.3 | 3500 | 1500 | 1 |  | 24017 |  | 68.6×68.6×68.6 | 62.6×62.6×62.6 |
| 0.4 | 3000 | 2000 | 1 |  | 27017 |  | 70.5 ×70.6×70.6 | 65.3×65.3×65.3 |
| 0.5 | 2500 | 2500 | 1 |  | 30017 |  | 73.6 ×73.6×73.6 | 67.8×67.8×67.8 |
| 0.6 | 2000 | 3000 | 1 |  | 33017 |  | 75.6×75.6×75.6 | 70.0×70.0×70.0 |
| 0.7 | 1500 | 3500 | 1 |  | 36017 |  | 78.5×78.6×78.6 | 72.1×72.2×72.2 |
| 0.8 | 1000 | 4000 | 1 |  | 39017 |  | 80.5×80.5×80.6 | 74.3×74.3×74.3 |
| 0.9 | 500 | 4500 | 1 |  | 42017 |  | 82.4×82.4×82.5 | 76.2×76.2×76.3 |
| 1.0 | 0 | 5000 | 1 |  | 45017 |  | 84.4×84.4×84.5 | 78.2×78.2×78.2 |
|  | | | | | | | | |

**Bulk-phase simulation data**

| **Table S3.** Data collected for the mixtures studied over 10 ns of bulk-phase simulation. Values are presented as mean with the standard deviation in parenthesis from a total set of 5000 data points. | | | | | | | | | | |
| --- | --- | --- | --- | --- | --- | --- | --- | --- | --- | --- |
|  |  |  |  |  |  |  |  |  |  |  |
|  |  |  |  |  |  |  |  |  |  |  |
|  | Vol-% EtOH^a^ |  | E_POT_ (kcal mol^-1^) | |  | Vol_Mix_ (Å^3^) | |  | Density (g cm^-3^) | |
| X_EtOH_ |  |  | -GUC | +GUC |  | -GUC | +GUC |  | -GUC | +GUC |
| 0 | 0 |  | -55806(113) | -55785(113) |  | 149889(264) | 150075(260) |  | 0.998(2) | 0.998(2) |
| 0.1 | 27 |  | -65807(124) | -65789(125) |  | 179855(331) | 180108(336) |  | 0.961(2) | 0.961(2) |
| 0.2 | 45 |  | -75687(130) | -75664(128) |  | 212051(371) | 212255(369) |  | 0.925(2) | 0.925(2) |
| 0.3 | 59 |  | -85600(134) | -85596(135) |  | 244677(428) | 244964(441) |  | 0.897(2) | 0.897(2) |
| 0.4 | 69 |  | -95583(142) | -95550(138) |  | 277492(477) | 277688(458) |  | 0.875(2) | 0.875(1) |
| 0.5 | 77 |  | -105602(145) | -105586(147) |  | 310416(495) | 310588(502) |  | 0.857(1) | 0.857(1) |
| 0.6 | 84 |  | -115702(150) | -115657(148) |  | 343514(545) | 343664(540) |  | 0.842(1) | 0.843(1) |
| 0.7 | 89 |  | -125862(156) | -125851(156) |  | 376667(563) | 376868(570) |  | 0.830(1) | 0.830(1) |
| 0.8 | 93 |  | -136120(157) | -136107(161) |  | 410021(579) | 410128(602) |  | 0.819(1) | 0.820(1) |
| 0.9 | 97 |  | -146422(161) | -146396(165) |  | 443724(583) | 443935(620) |  | 0.810(1) | 0.810(1) |
| 1.0 | 100 |  | -156655(165) | -156625(165) |  | 478024(634) | 478241(631) |  | 0.800(1) | 0.800(1) |
| ^a^ Estimated using a molecular volume of ethanol (EtOH) of 95.6048 Å^3^ (478024 Å^3^/5000 molecules) | | | | | | | | | | |
|  |  |  |  |  |  |  |  |  |  |  |

**Excess volume**


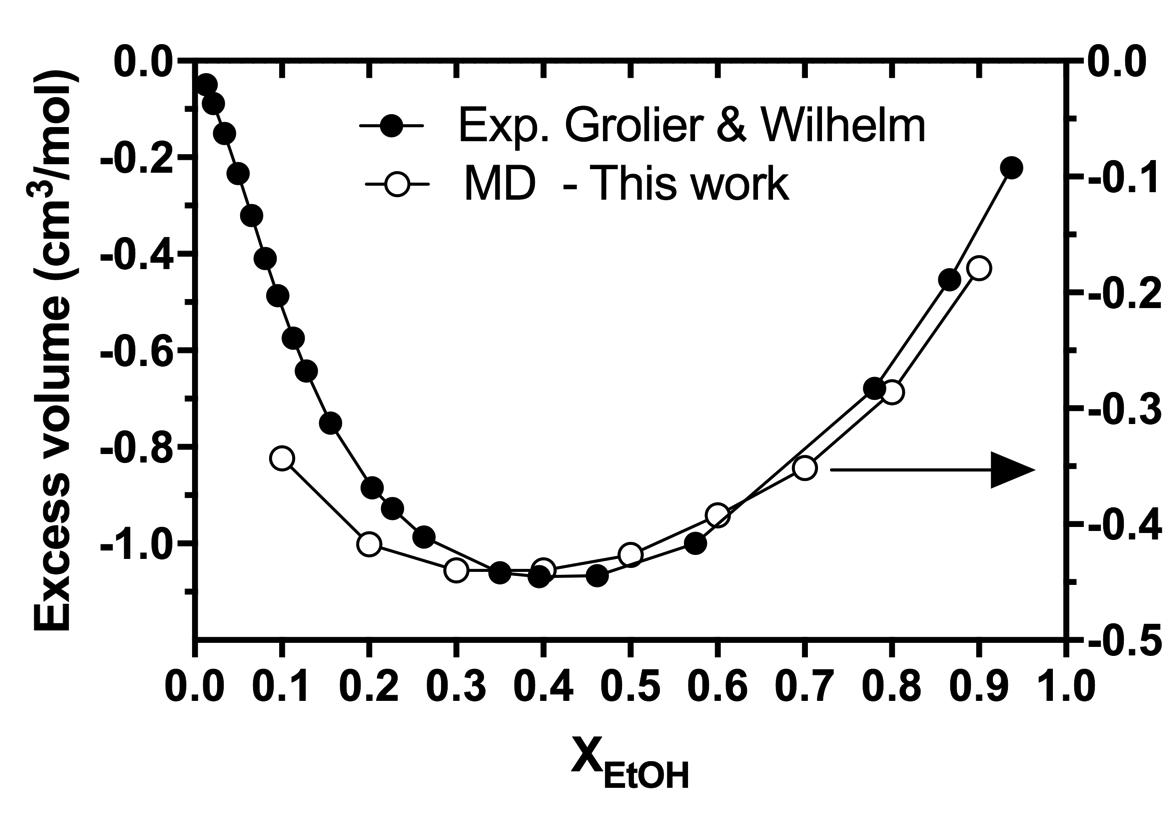


**Figure S1.** Excess volumes calculated for the series of ethanol-water bulk-phase mixtures studied in the absence of the solute, guaiacol. Reference data are taken from Grolier, J-P. E., Wilhelm, E. Excess volumes and excess heat capacities of water + ethanol at 298.15 K. *Fluid Phase Equilib*. **6**, 283-287 (1981). Note that the simulated data (values associated with the right-hand side y-axis) underestimated the interactions between ethanol and water molecules in the mixture however (with a factor of 3) show qualitative agreement with experimental data.

**Static dielectric constant**


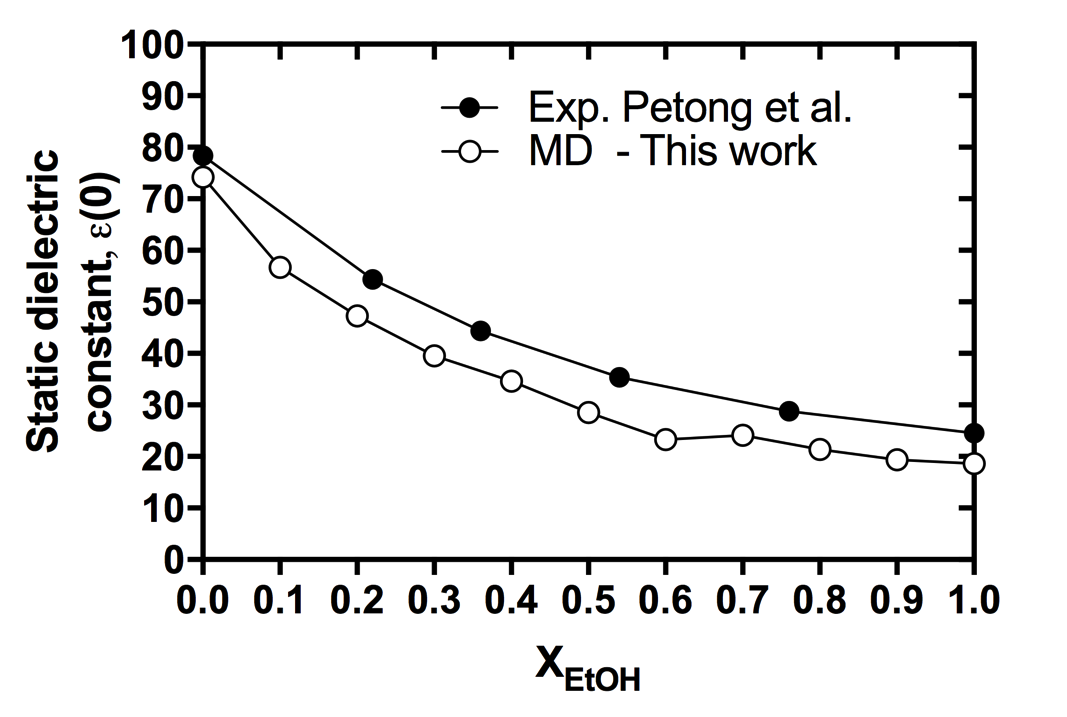


**Figure S2.** Static dielectric constants calculated for the series of ethanol-water bulk-phase mixtures studied in the absence of the solute guaiacol. Reference data are taken from Petong, P., Pottel, R., Kaatze, U. Water−ethanol mixtures at different compositions and temperatures. A dieletric relaxation study. *J. Phys. Chem. A* **104***,* 7420-7428 (2000)*.*

**Solvent self-diffusion rates**

| **Table S4.** Data collected for the mixtures (with or without the solute guaiacol (GUC) studied over 10 ns (bulk) or 50 ns (liquid-air interface, LAI) of simulation. Values are presented as mean with the standard deviation in parenthesis from at total of 5000 data points. During linear fitting of data, R^2^ was always > 0.997. | | | | | | | | | |  |
| --- | --- | --- | --- | --- | --- | --- | --- | --- | --- | --- |
|  |  |  |  |  |  |  |  |  |  |  |
|  |  |  |  |  |  |  |  |  |  |  |
|  |  |  | Self-diffusion, *D /* 10^-5^ cm^2^ s^-1^ | | | | | | |  |
|  | Vol%  EtOH^a^ |  | Water | | |  | EtOH | | |  |
| X_EtOH_ |  |  | -GUC | +GUC | +GUC  (LAI)^b^ |  | -GUC | +GUC | +GUC  (LAI)^b^ |  |
| 0 | 0 |  | 2.90(0.1) | 2.90(0.0) | 2.81(0.0) |  | - | - | - |  |
| 0.1 | 27 |  | 2.03(0.1) | 2.09(0.0) | 2.02(0.0) |  | 1.23(0.1) | 1.15(0.0) | 1.99(0.0) |  |
| 0.2 | 45 |  | 1.61(0.1) | 1.59(0.0) | 1.58(0.0) |  | 0.94(0.1) | 0.95(0.0) | 1.42(0.1) |  |
| 0.3 | 59 |  | 1.37(0.1) | 1.37(0.0) | 1.39(0.0) |  | 0.88(0.0) | 0.91(0.0) | 1.24(0.1) |  |
| 0.4 | 69 |  | 1.16(0.1) | 1.17(0.0) | 1.12(0.0) |  | 0.83(0.1) | 0.85(0.0) | 1.34(0.1) |  |
| 0.5 | 77 |  | 1.09(0.0) | 1.14(0.0) | 1.10(0.0) |  | 0.85(0.0) | 0.85(0.0) | 1.20(0.0) |  |
| 0.6 | 84 |  | 1.02(0.0) | 1.04(0.0) | 1.18(0.0) |  | 0.84(0.0) | 0.87(0.0) | 1.18(0.1) |  |
| 0.7 | 89 |  | 0.99(0.0) | 0.97(0.0) | 1.09(0.0) |  | 0.85(0.0) | 0.83(0.0) | 1.23(0.1) |  |
| 0.8 | 93 |  | 1.05(0.0) | 0.98(0.0) | 0.42(0.0) |  | 0.85(0.0) | 0.84(0.0) | 0.49(0.0) |  |
| 0.9 | 97 |  | 0.98(0.0) | 0.98(0.0) | 0.43(0.0) |  | 0.84(0.0) | 0.87(0.0) | 0.53(0.0) |  |
| 1.0 | 100 |  | - | - | - |  | 0.85(0.0) | 0.86(0.0) | 0.53(0.0) |  |
| ^a^ Estimated using a molecular volume of ethanol (EtOH) of 95.6048 Å^3^ (478024 Å^3^/5000 molecules).  ^b^ Liquid-air interface simulations | | | | | | | | | | |
|  |  |  |  |  |  |  |  |  |  |  |

**Bulk-phase spatial distribution functions**


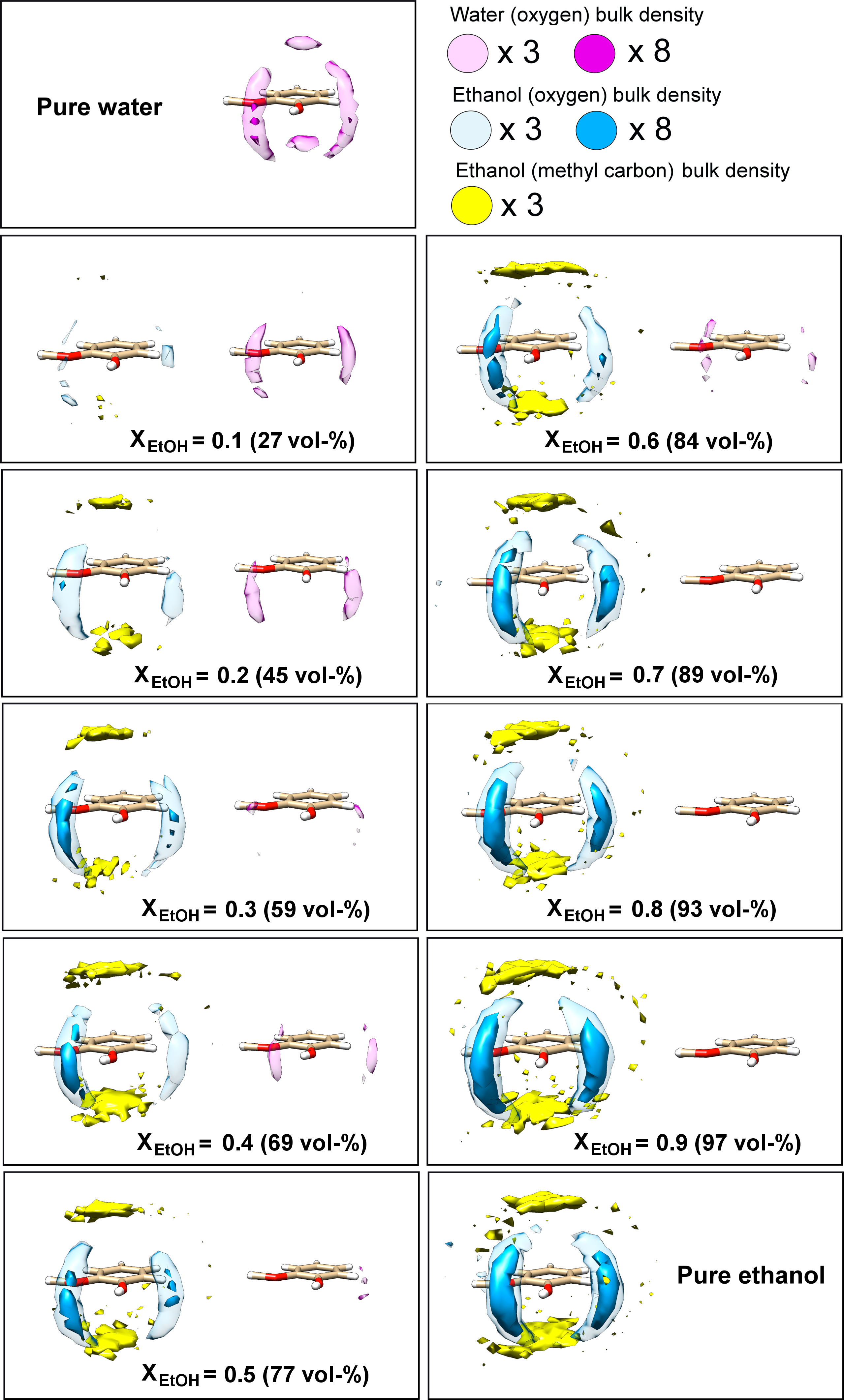


**Figure S3.** Atomic spatial distribution functions (SDFs) of water oxygen and EtOH oxygen and methyl carbon components around a single guaiacol molecule in bulk-phase mixtures of varying EtOH content. In each panel, the water and EtOH guaiacol solvation patterns are shown on the right- and left-hand side, respectively. For better visibility, the atomic densities of selected atoms of the liquids, *i.e.* water and ethanol oxygen, and the methyl carbon of ethanol, are presented using either three or eight times the water or ethanol bulk densities.

**Maximum bulk-phase solvent atomic density**


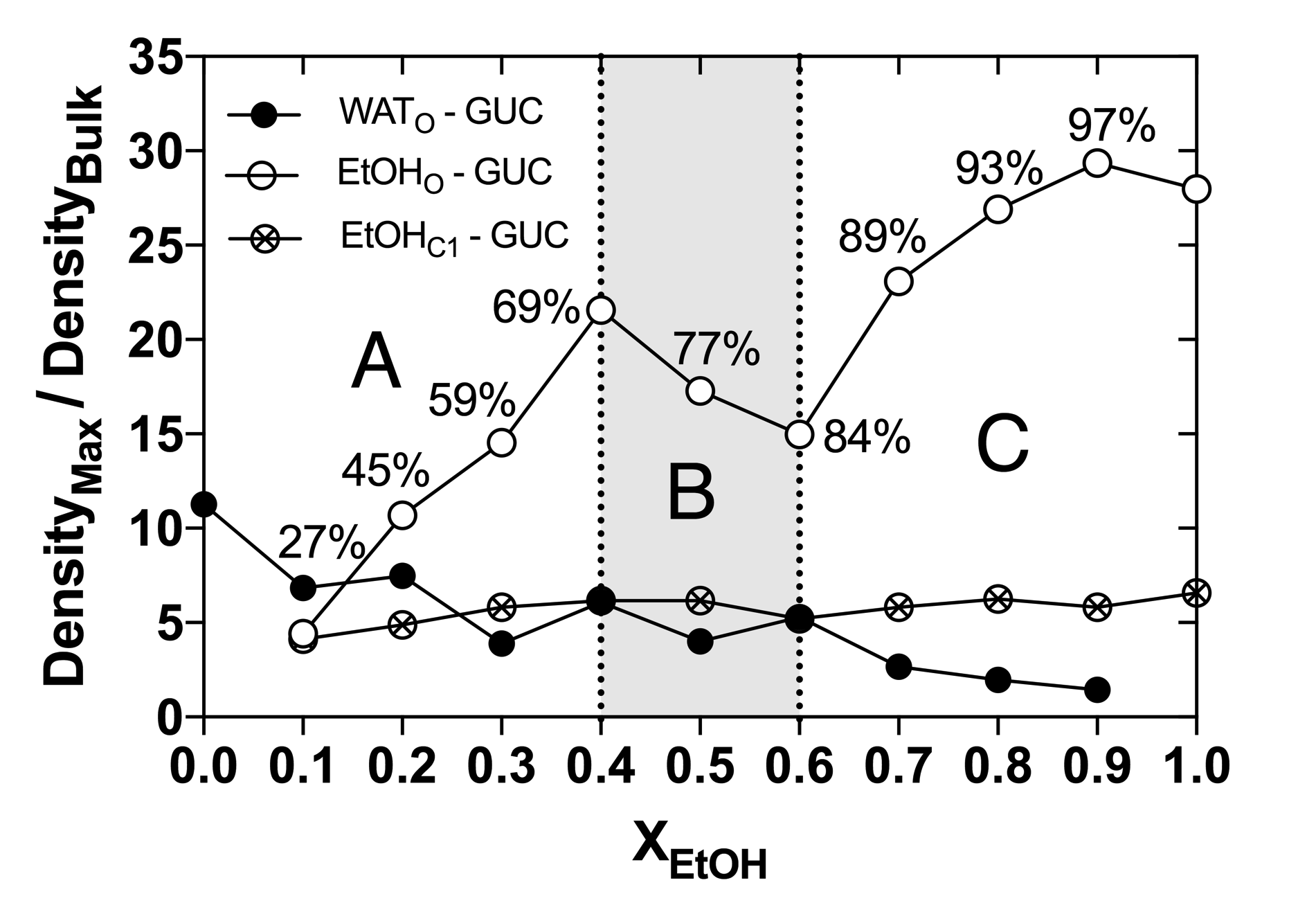


**Figure S4.** Maximum occupancies (Density_Max_/Density_Bulk_) for the components of the solvents around guaiacol (GUC). The maximum atomic density of the water (filled circles) and EtOH (open circles) oxygen atoms, WAT_O_ and EtOH_O_, respectively, and the EtOH methyl carbon, EtOH_C1_, (crossed circles) were calculated on the basis of the spatial distribution functions presented in **Figure S3**. As a guide for the eye, the dots are connected with lines. Ethanol concentration ranges of interest are marked out with the letters A-C.
